# Ionic Liquid-Assisted Selective Extraction and Partitioning of Biomolecules from Macroalgae

**DOI:** 10.1021/acssuschemeng.2c05823

**Published:** 2023-01-24

**Authors:** Edgar Suarez Garcia, Carlota F. Miranda, M. Teresa Cesario, Rene H. Wijffels, Corjan van den Berg, Michel H. M. Eppink

**Affiliations:** † Bioprocess Engineering, AlgaePARC, Wageningen University and Research, P.O. Box 16, 6700 AAWageningen, The Netherlands; ‡ IBB-Institute for Bioengineering and Biosciences, Bioengineering Department, Instituto Superior Técnico, Av. Rovisco Pais, 1049-001Lisboa, Portugal; § Nord University, Faculty of Biosciences and Aquaculture, N-8049Bodø, Norway

**Keywords:** macroalgae, selective extraction, ionic liquids, proteins, carbohydrates, solvent recovery, ultrafiltration

## Abstract

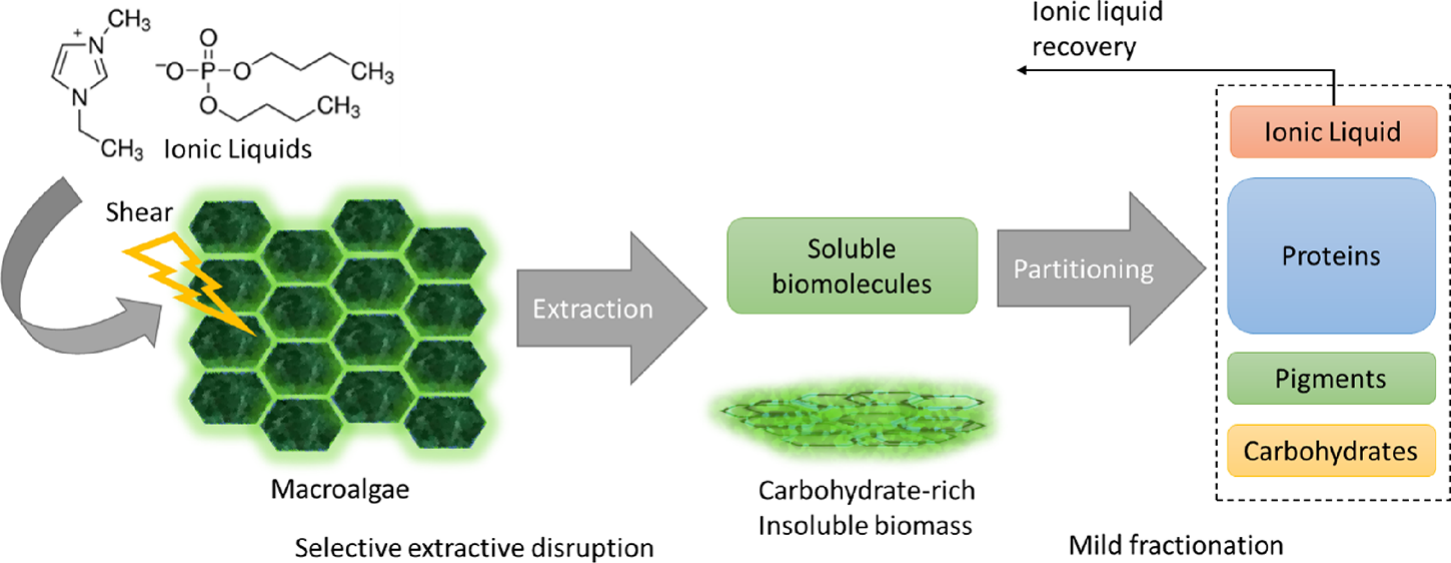

Macroalgae are a promising feedstock for several industries due to their large content of proteins and carbohydrates and the high biomass productivities. A novel extraction and fractionation concept based on ionic liquids (ILs) using *Ulva lactuca* as model organism is presented. Biomolecules are first extracted by means of IL-assisted mechanical shear, followed by two-phase partitioning or ultrafiltration in order to fractionate proteins and carbohydrates and to recover the IL. Ethyl methyl imidazolium dibutyl phosphate ([Emim][DBP]) is strongly selective to proteins, leading to extraction yields up to 80.4% for proteins and 30.7% for carbohydrates. The complete process, including extraction and ultrafiltration, allowed protein recovery of up to 64.6 and 15.4% of the carbohydrates in the retentate phase, while a maximum of 85.7% of the IL was recovered in the permeate phase. The native structure of the extracted proteins was preserved during extraction and fractionation as shown by gel electrophoresis. Selective extraction of proteins from macroalgae under non-denaturing conditions using ILs followed by the recovery of IL using ultrafiltration is for the first time reported. The proposed extraction–fractionation approach is simple and can be potentially applied for the biorefinery of macroalgae at the commercial scale.

## Abstract 2

Protein recovery from marine organisms (macroalgae) under non-denaturing conditions with reusable green solvents strengthens sustainability.

## Highlights

- Ionic liquid-mediated biorefinery of *Ulva lactuca* is demonstrated.
- [Emim][DBP] can selectively extract proteins from the algal biomass.
- Up to 80% of the proteins could be extracted under mild conditions.
- Ultrafiltration was implemented to recover ionic liquids from the algal extracts.

## Introduction

The global demand for biomolecules is rising. For food applications alone, the need for proteins is projected to increase from up to 32 to 78% of the present value of 202 tons per year depending on the consumers’ behavior and population growth.^1^ This accelerated increase also escalates the demand for feed, materials, and fuels, leading thus to the challenge of finding sustainable feedstocks and developing novel transformation processes to convert raw materials into products, ingredients, and intermediates.

Marine algae have long been suggested to be an ideal sustainable feedstock for biorefineries^2^ due to their superior productivities compared to traditional crops, no direct competition for fresh water and arable land, no seasonal dependence, and a rich and diverse composition.^3^ Marine algae can be classified into two main groups: microalgae (unicellular organisms) and macroalgae (multicellular species). Likewise, macroalgae are grouped in three categories depending on the main pigments present in each strain: green, brown, and red algae.^4^ In general, macroalgae lack lignin and hemicellulose and are rich in polysaccharides, minerals, and proteins. In addition, these organisms show higher volumetric productivities and biomass densities compared to microalgae^5^ and thus constitute a very attractive lipid-free biorefinery platform. Macroalgal products find applications in the fields of biomaterials,^6^ biofuels,^7^ environmental remediation,^8^ feed,^9,10^ food, and pharma.^11,12^ Proteins from macroalgae display an interesting range of functional properties,^13^ and the amino acid profile is comparable to commercial protein isolates,^10^ which make them particularly interesting for food products.

The extraction of proteins from green macroalgae poses a major challenge as proteins are often embedded in a complex matrix of polysaccharides.^14^ The most common processes reported in the literature include extraction in aqueous and alkaline media,^10,15^ mechanical shear,^16,17^ and enzymatic treatments.^15,17,18^ Such processes, however, result in modest extraction yields or can compromise the functional properties of the biomolecules when harsh conditions are used. Furthermore, the fractionation of biomolecules after the extraction step has only been reported by means of aqueous two phase systems containing PEG and salts.^15,19^ The development of novel fractionation methods that ensure mild processing and high extraction yields is crucial to promote the commercialization of algal products.

In recent years, the unique physicochemical properties of ionic liquids (ILs) have attracted the attention of industry and academia to be applied as green solvents for the dissolution of lignocellulosic biomass components,^20^ the extraction of biomolecules from several feedstocks,^21^ and the fractionation, under mild and stable conditions, of model proteins^22^ and biomolecules from microalgae.^23^ The implementation of ILs for macroalgae, however, has been limited to a handful of studies where algal biomass is directly treated at high temperatures (90–160 °C) and long contact times (3–360 min) to release carbohydrates.^24−27^ To the authors’ knowledge, mild extraction of proteins using ILs has only been investigated recently to recover phycobiliproteins from the red macroalga *Gracilaria* sp.^28^

One of the main hurdles for the implementation of ILs in commercial processes is their high cost^29^ and uncertainty regarding their toxicity. High costs can be circumvented by synthesizing novel ILs from cheap sources^30^ and by developing technologies that allow their recovery and reuse. Several methods are reported in literature for the recyclability of ILs, including phase induction, adsorption, extraction, and membrane processes.^31^ Pervaporation, electrodialysis, nanofiltration, and reverse osmosis are commonly reported in model systems.^31^ Despite its widespread implementation, to the authors knowledge ultrafiltration has not been investigated for the recovery of ILs from algae extracts.

In this investigation, the extraction and fractionation of proteins and carbohydrates from the green macroalgae *Ulva lactuca* was addressed. The proposed approach is based on the integration of the disruption and extraction steps by means of bead milling and chemical solubilization mediated by ILs. Furthermore, biomolecule fractionation is studied using two methods: induced phase separation and ultrafiltration. The latter is also proposed in order to recover IL from the algal extract. All investigated processes are simple and are conducted under mild conditions, which is a unique contribution of the present work.

## Material and Methods

### Chemicals

All chemicals used in this investigation were of analytical grade. Chloroform and methanol were obtained from Biosolve. Phosphate saline buffer (PBS) pH 7 was prepared by mixing 0.21 g of KH_2_PO_4_, 0.48 g of Na_2_HPO_4_·2H_2_O, and 9.00 g of NaCl in 1 L of distilled water. NaOH, KH_2_PO_4_, Na_2_HPO_4_·2H_2_O, NaCl, and K_2_HPO4 were purchased from Merck Millipore. Concentrated phenol, concentrated sulfuric acid, polyethylene glycol (PEG1000), and Na_2_CO_3_ were obtained from Sigma-Aldrich. The ionic liquids 1-butyl-3-methylimidazolium acetate (98%) [Bmim][Ac], 1-ethyl-3-methyl-imidazolium dibutyl phosphate (97%) [Emim][DBP], 1-butyl-3-methylimidazolium dibutyl phosphate (98%) [Bmim][DBP], 1-butyl-3-methylimidazolium chloride (98%) [Bmim][Cl], and choline chloride (98%) [Ch][Cl] were acquired from Iolitec GmbH (Germany).

### Macroalga Collection and Pretreatment

*U. lactuca* was kindly provided by Dr. Willem de Visser from Wageningen Plant Research. This alga was cultivated in 1 m^3^ tanks containing filtered sea water (Oosterschelde, The Netherlands) at the greenhouse facilities of Wageningen University (Nergena, Wageningen, The Netherlands). The seawater was replaced on a monthly basis. Samples were collected every month from these culture tanks (Nergena, Wageningen University and Research) in the period of June–August. After biomass collection, the excess water was removed and the samples were freeze-dried in a 2 × 3 × 3 sublimator (Zirbus Technology GmbH, Germany) for 72 h. Dried samples were ground to a particle size of ∼0.5 mm using a kitchen coffee grinder, stored in sealed bags, and maintained in the dark at room temperature until further use.

### Conventional Extraction Methods

Several extraction methods commonly reported in literature were investigated in this report. In accordance with Fleurence et al.,^15^ aqueous extraction was conducted by gentle mixing of algal biomass in MilliQ water (1:20 w/v) for 12 h at 4 °C. Samples were centrifuged at 10,000*g* for 20 min, and the supernatant and pellet were collected for analysis and alkaline extraction, respectively. Pellets were resuspended in 0.1 M NaOH and stirred for 1 h at room temperature. Samples were centrifuged at 10,000*g* for 20 min, and the supernatants were collected for analysis. High-shear extraction was done in accordance with Harnedy and FitzGerald^32^ and Postma et al.^17^ Algal biomass was suspended in distilled water, and the pH was adjusted to 8. Next, the suspension was stirred at 6400 rpm for 10 min using a rotor-stator disperser (Ultra-Turrax T25, IKA, Germany). The suspension was maintained in an ice-water bath to prevent overheating. Samples were centrifuged at 10,000*g* for 20 min, and the supernatants were collected for analysis. Aqueous two phase extraction was applied based on the process reported by Jordan and Vilter^19^ and Fleurence et al.^15^ The phase-forming components (9.9 wt % PEG and 10.9 wt % Na_2_CO_3_) were dissolved in MilliQ water, followed by the addition of algal biomass (3 wt %) and strong mixing for 20 min. Next, samples were centrifuged at 4500*g* for 20 min to accelerate two-phase formation. The volumes of each phase were determined and analyzed for protein and carbohydrate content.

### Theoretical Properties of the Ionic Liquids

The theoretical hydrogen bond basicity (HBB) of the anion and hydrophobicity of the anions and cations of the ionic liquids investigated in the present study were estimated from the linear free energy relationship parameters published by Cho et al.^33^ Due to the lack of descriptors, theoretical and experimental data for the anion dibutyl-phosphate, the descriptors for the anion diethyl-phosphate, were used.

### Ionic Liquid-Assisted Extraction

**Figure 1:**
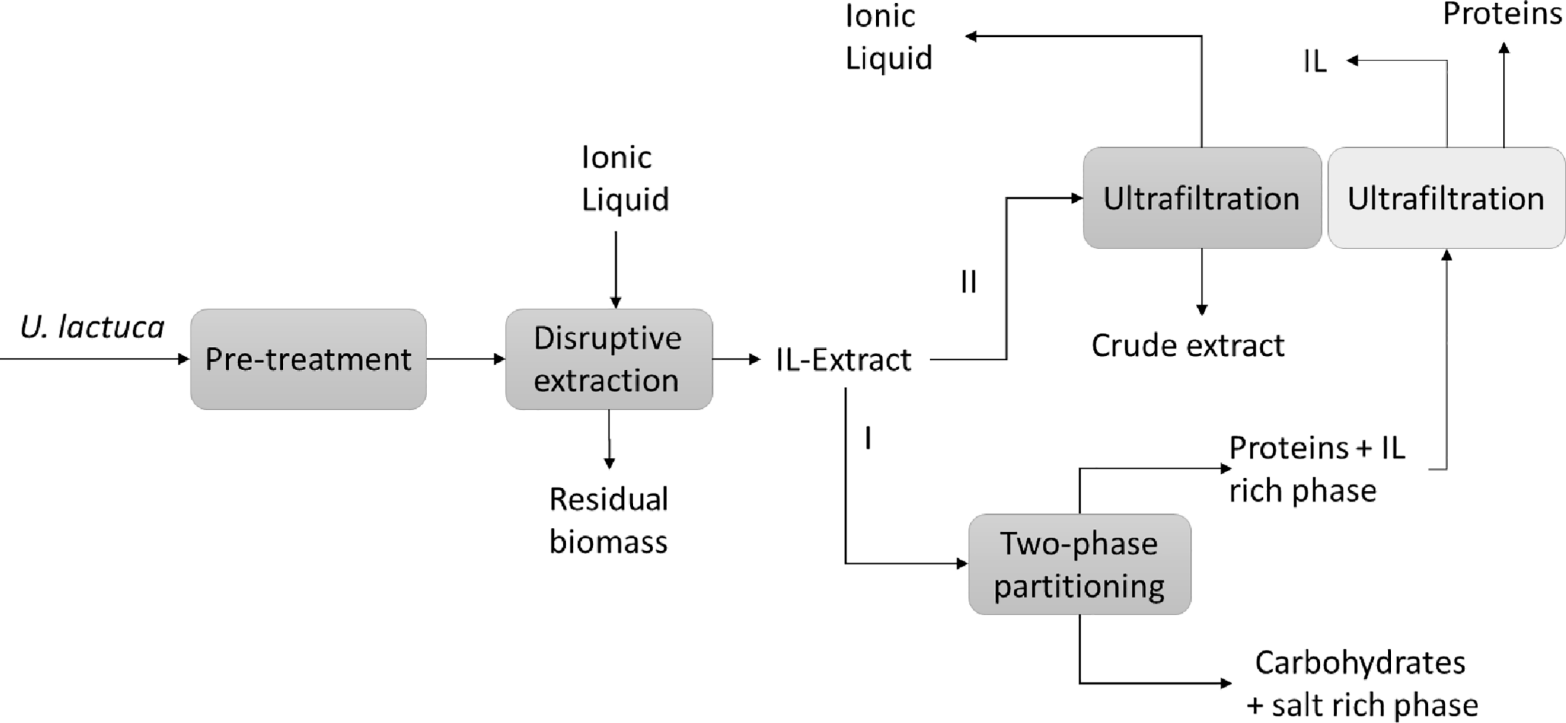
Block diagram for the IL-mediated extraction process, followed by IL recovery or phase partitioning and subsequent IL recovery via ultrafiltration.

An overview of the IL-assisted extraction and fractionation process investigated in the present work is shown in Figure 1. To summarize, five ILs were screened in the extractive-disruption step (bead milling + IL). The best-performing IL was chosen for two-phase partitioning for biomolecule fractionation (route I)) and/or ultrafiltration for IL recovery (route II).

Phase partitioning was attained by the addition of a salt-rich phase to separate carbohydrates from proteins (Figure 1, route I), and then the IL could be recovered by ultrafiltration.

To test the performance of the ILs for the extraction of biomolecules from *U. lactuca*, the extraction yields for proteins and carbohydrates were measured after contacting directly algal biomass with solutions containing 40 wt % IL in bead beating tubes (lysis matrix D, MP Biomedicals, USA). The samples were subjected to intense mixing using a Precellys 24 Homogenizer (Bertin Instruments, France) for three cycles of 60 s each at 6500 rpm and a 120 s pause in between cycles to avoid overheating (total extraction time ≈ 10 min, *T* = 25 °C ± 2 °C). The resulting suspensions were centrifuged for 10 min at 3000*g* and 4 °C, and the supernatant was collected for analysis. The IL leading to the highest extraction yields was chosen for further experiments. The effect of the concentration of the best performing IL on the extraction yields was investigated in the range of 0–40 wt % (IL). At higher concentrations, handling and processability becomes difficult due to the viscosity of the IL. In addition, interference with the analytical methods becomes significant. For all the studies with ILs, a biomass load of 1 wt % was used. Moreover, proper controls were used, and the effect of the IL on the quantification methods was carefully considered. Samples containing only IL solutions were used as controls, and the corresponding blanks were used for the data analysis.

### Two-Phase Partitioning

To test the performance of phase induction for the partitioning of extracted biomolecules, the salt K_2_HPO_4_ was selected as a second phase-forming component. In order to determine the operational points, the binodal curve was prepared according to the cloud point titration method.^34^ In short, the salt-rich solution is added dropwise to the IL-rich solution followed by mixing and settling until a cloudy region is observed, indicating the two-phase region. Partitioning experiments were performed by strong mixing of the algae extract containing IL with a known amount of salt, followed by a spontaneous phase split. Samples from the top and bottom phases were taken for the quantification of proteins and carbohydrates.

### Biomolecule Fractionation and Ionic Liquid Recovery

Ultrafiltration experiments were carried out with two different membrane systems. An ultrafiltration stirred cell (Model 8050) with a 10 kDa polyether sulfone membrane (Biomax) was operated to reach a 5× concentration factor at a constant transmembrane pressure of 2.2 bar and filtrate flow of 0.1 mL min^–1^. A 3 kDa centrifugal filter (Ultracell) was run for 40 min at 4000*g* to obtain a 4× concentration factor. All filter materials were purchased from Merck Millipore, USA. The retentates and permeates were collected and analyzed for protein and carbohydrate content. Control experiments were conducted with aqueous solutions containing only IL under the same flow and centrifugation conditions as described for the algal extracts. The resulting permeate and retentates were analyzed for total carbohydrates in order to verify if the IL solubilizes the membrane’s polysaccharides.

### Biomass Characterization and Quantification

Dry weight was estimated gravimetrically after drying a known amount of biomass in a convection oven (Nabertherm, Germany) to constant weight at 100 °C. Total ash was determined after burning a known amount of algal biomass in a furnace (L 24/11, Nabertherm, Germany) at 575 °C and regarding the remaining material as total ash. Total lipids were quantified according to the method of Folch.^35^ In short, five extraction cycles were conducted on dry biomass using a mixture chloroform:methanol:PBS 2:1:0.8 V V^–1^. The solvent phase was collected after every step, and the excess solvent was evaporated using a vacuum concentrator (RVC 2-25 CDplus, Christ, Germany). The remaining material was weighted and regarded as total lipids.

The methods of Lowry^36^ and Bradford^37^ were implemented for the quantification of protein in the algal biomass. However, significant interference of the IL with the method of Lowry prevented its further use. On the contrary, negligible interference of the ILs with method of Bradford was observed, and therefore, it was used for the quantification of proteins in all samples. Measurements were conducted with a commercial kit (Pierce Coomassie Protein Assay, Thermo Fisher Scientific, USA), using bovine serum albumin (Sigma-Aldrich) as a protein standard and ILs solutions as blanks. Bradford is a colorimetric method that correlates protein content to the absorbance shift of the dye Coomassie Blue, which can be quantified at 595 nm. Total carbohydrates were determined with the method of Dubois,^38^ which is based on the colorimetric reaction of carbohydrates and phenol in concentrated sulfuric acid, which can be measured at 483 nm. Glucose (Sigma-Aldrich) was used as a carbohydrate standard. Absorbances at 595 and 483 nm were measured with a microplate reader (Infinite M200, Tecan, Switzerland).

Quantification of the IL for the filtration experiments was conducted by ultrahigh-performance liquid chromatography (UHPLC Nexera X2, pump LC-30 AD, autosampler SIL-30 AC, Refractive Index Detector RID-20A, Shimadzu, USA) using a Rezex ROA-Organic Acid column coupled with a security guard (300 mm × 7.8 mm, Phenomenex, USA). Prior to analysis, all samples were centrifuged at 4500*g* for 20 min to remove suspended particles. Samples were injected (20 μL) and run at 0.6 mL min^–1^ under an isocratic mode with 0.005 N H_2_SO_4_ as a mobile phase. The column was kept at 60 °C under a pressure of 55 bar. Solutions of IL in MilliQ water were used as standards.

Mass yields (*Y*) per component (*i*) were estimated according to eq 1:
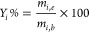
1

Here, *m_i,e_* is the mass of component *i* (protein, carbohydrates, IL) in the extract phase and *m_i,b_* is initial mass of component *i* before the extraction or fractionation step. All calculations are conducted on a dry-weight basis.

### Acrylamide Native Gel Electrophoresis

Protein samples were diluted with native buffer at a ratio 1:0.8 V V^–1^. Twenty-five microliters of the resulting solution was loaded per lane in a 4–20% Criterion TGX gel. Electrophoresis was run at 125 V for 75 min using tris-glycine as running buffer. All materials were procured from BioRad. Gels were stained using the Pierce Silver Stain Kit (Thermo Fisher Scientific, USA). Gel images were acquired with an ImageScanner III (GE Healthcare, UK).

### Statistics

Unless otherwise noticed, all experiments were conducted in triplicate. The data is presented as mean values and the corresponding standard deviation. The variation of the experimental data under different treatments was analyzed by one-way analysis of variance (ANOVA) at 95% confidence level. When significant differences were found (i.e., *p* < 0.05), the Tukey’s Honest Significant Difference test (HSD) was used to detect significant differences between specific treatments. All analysis were performed in R (v3.4.0).

## Results and Discussion

### Biomass Characterization

*U. lactuca* is a green macroalga that occurs in several coastal benthic areas around the world, which implies that its biochemical composition varies greatly depending on the cultivation conditions and harvesting season. In general, for green macroalgae, the content of carbohydrates ranges from 25 to 72%,^39,40^ proteins range from 3 to 35%,^9,39,41^ minerals range from 10 to 31%, and lipids can reach up to 4.3%.^40^

The biomass under investigation showed a protein content of 17.8 ± 0.8% (dw) using the method of Lowry and 3.7 ± 0.3% with the method of Bradford. The method of Bradford has been reported to significantly underestimate the protein content in algae due to the presence of free amino acids and small peptides, which are less reactive in the Bradford assay.^16,42^ Nonetheless, this method is less likely to be affected by non-protein compounds found in marine algae.^42^ Due to the interference of the method of Lowry with the ionic liquids used in this study, all protein analysis and calculations are based on the method of Bradford.

The carbohydrate content reached 54.9 ± 1.2%, the ash content reached 22.8 ± 0.3%, and the lipid content reached 4.9 ± 0.3% (dw). All the experimental compositions are in good agreement with the reported values for *U. lactuca.*(43) The protein content, amino acid profile, and carbohydrates and fatty acids present in *U. lactuca* make it an interesting organism not only as source of chemical building blocks and fuels but also as a functional ingredient in food products.^44^

### Extraction of Biomolecules with Conventional Methods

In general, the first step in the extraction and fractionation of biomolecules from macroalgae is the disintegration of the cell wall and consequent release of the intracellular content (cell lumen). The cell wall of *U. lactuca* is composed of an intricate arrangement of polysaccharide layers and embedded proteins interacting by means of hydrogen bonds and ionic forces.^14^ Such a cell envelope poses a major hurdle to ensure mild processing and to reach high extraction yields. In this regard, several technologies have been reported in literature for the biorefinery of green seaweeds, including physical, chemical, and thermal treatments.^10^

**Figure 2:**
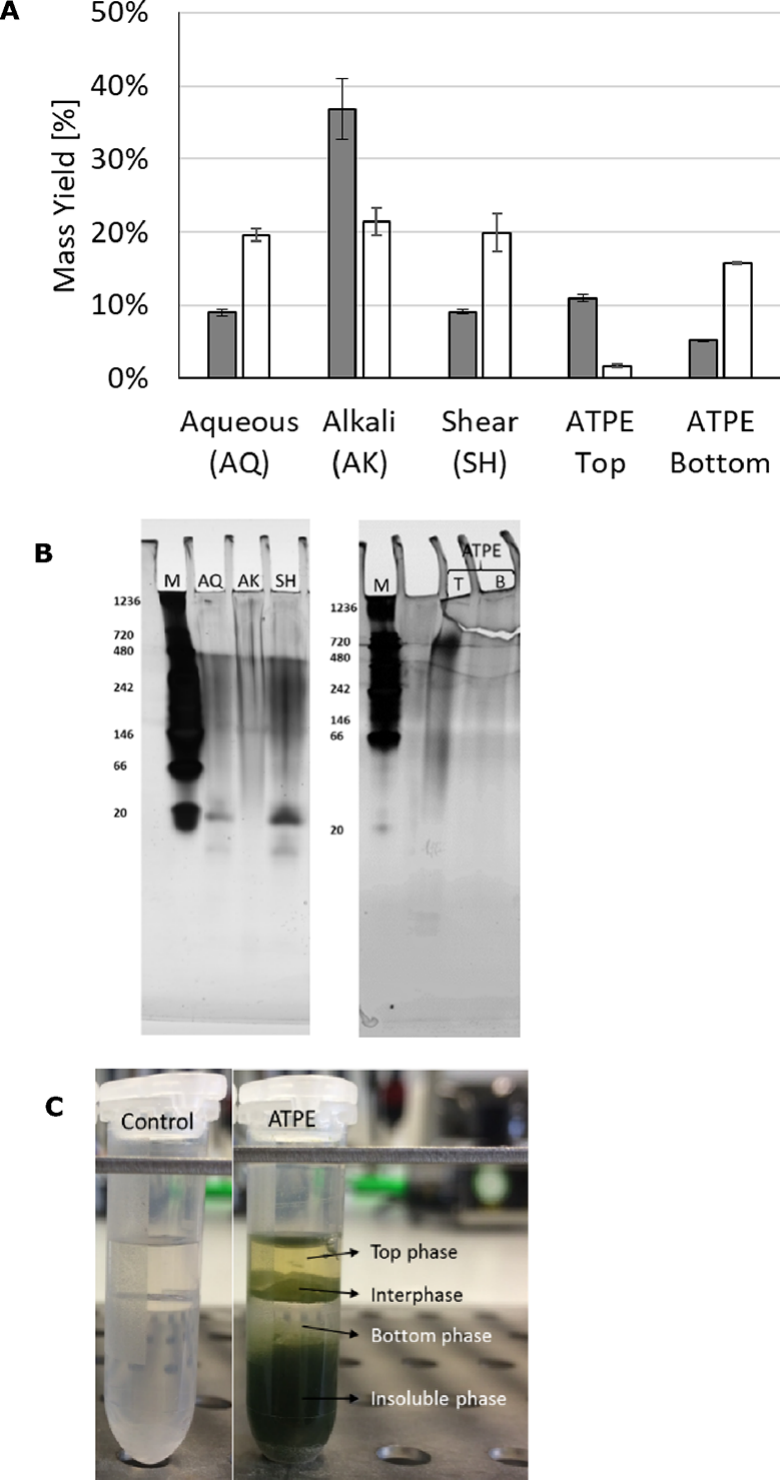
(A) Extraction yields for proteins (gray bars) and carbohydrates (white bars) for samples after conventional extraction methods or ATPE. Data correspond to average values and corresponding standard deviation as error bars (*n* = 3). (B) Native gel electrophoresis (M: Marker, T: Top, B: Bottom; white arrows indicate main protein bands). (C) Four-phase system developed after ATPE.

Due to the simplicity, low costs, and reported yields, four conventional processes were evaluated in the present investigation: osmotic shock (Aqueous), alkali solubilization (Alkali), high shear disruption (Shear), and aqueous two-phase extraction (ATPE). The corresponding extraction yields for proteins and carbohydrates are shown in Figure 2A. The highest protein yield is obtained by means of alkali solubilization (∼36%), while the other methods resulted in statistically equal yields of ∼10% (*p* > 0.05). At alkaline pH, the extraction of proteins increases mainly because of the higher solubility of algal proteins above their isoelectric point and due to the interaction of NaOH with the structure of cellulose, one of the main constituents of the cell wall of *U. lactuca.*(14) The Na^+^ cations penetrate the intra-crystalline spaces of cellulose, causing its swelling^45^ and thus favoring the release of biomolecules from the cell lumen. Further increments in pH can enhance protein yields at the expense of cellulose solubilization and protein hydrolysis; however, under such conditions, the functionality of the proteins will be compromised. To our surprise, shear processing did not result in higher yields. This was probably due to the pretreatment conducted on the harvested alga (drying and milling), which made the rotor-stator system ineffective of interacting with the biomass particles.

Regarding carbohydrates, all the methods resulted in statistically equal yields of 16–21% (*p* > 0.05). This can be explained considering that ulvan, the second main polysaccharide in *U. lactuca*, is water-soluble and therefore easily extracted under most conditions tested. Although the two-phase extraction process did not provide significant improvements in the extraction yields, the method is particularly attractive as it allows the selective partitioning of proteins, pigments, and carbohydrates. Figure 2A shows that proteins are primarily extracted in the more hydrophobic top phase (PEG-rich), while carbohydrates are mainly present in the hydrophilic bottom phase (salt-rich). This confirms that the anionic or neutral polysaccharides from *U. lactuca* display a strong affinity for the salt-saturated phase, while proteins show affinity for the PEG phase.^15^ Another interesting aspect of the ATPE process is the formation of a four-phase system, which was not described by the reference authors. In our investigation, two additional layers developed (Figure 2C): the interphase and insoluble phase (pellet). The characterization of the interphase was not conducted due to its size but it might contain macromolecular complexes, which display an intermediate polarity between PEG and Na_2_CO_3_. From Figure 2C, it is also clear that pigments are extracted almost exclusively in the top phase.

The native gel electrophoresis patterns (Figure 2B) revealed that under the aqueous and shear treatments, mild conditions prevail as more protein bands are observed, whereas for the alkali and ATPE process, there is significant effect on the proteins as no clear protein bands are observed. The low-molecular weight protein bands disappear for samples treated at pH 12, suggesting protein hydrolysis or precipitation. Loss of low-molecular weight proteins also takes place during ATPE. In addition, a strong band at ∼700 kDa is developed in the top phase of ATPE, suggesting protein aggregation. Fleurence et al., (1995),^15^ also reported the absence of protein bands for *Ulva* sp. extracts containing PEG but did not provide further explanation on their findings.

The experimental protein yields are in good agreement with Fleurence et al.,^15^ who reported values of 9.4–13.8% for aqueous, 17.5–25.2% for alkali, and 19.1–31.1% for ATPE extractions on *Ulva rigida* and *Ulva rotundata*. The authors published additional data for sonication (10.3–16.1% yield) and enzyme treatments (22–25% yield) and concluded that alkali and ATPE extraction were the best methods. Postma et al.^17^ reported the yield of proteins from *U. lactuca* using aqueous extraction (19.5%), high-shear homogenization (39.1%), pulsed electric fields (15.1%), and enzyme hydrolysis (26.1%). The extraction yields for carbohydrates were 44.7, 51.3, 14.8, and 28.1%, respectively. The notably higher yields reported by Postma et al. compared to the present work can be attributed to variations in the extraction protocols (e.g., 24 h instead of 12 h, 30 °C instead of 4 °C) and strain-related differences derived from the culture conditions,^32^ harvesting season, and biomass pre-treatment.

### Ionic Liquid-Assisted Extractive Disruption and Partitioning

In order to enhance the extraction yields of biomolecules from macroalgae, it is necessary to fully disentangle the cell wall structure and organelles under mild non-denaturing conditions. As the content of cellulose in the cell wall of *U. lactuca* reaches up to 70 wt %,^46^ several ionic liquids (ILs), which can potentially solubilize cellulose, were screened. ILs are poorly coordinated salts, which are liquid at room temperature. Due to their unique chemical versatility, ILs have been extensively investigated in chemical synthesis, catalysis, and electrochemistry as well as for the processing of woody biomass.^20^

The mechanism of solubilization of cellulose by ILs has been investigated in detail. Both the cation and anion of the IL are believed to form electron donor–acceptor complexes with the oxygen and hydrogen atoms of the cellulose. Such interactions cause the separation of the hydroxyl groups from the cellulose chains, which ultimately results in their solubilization.^47^ ILs that solubilize cellulose are in general characterized by high values of the solvents’ hydrogen-bond basicity.^20^ In addition, experimental evidence indicates that the anion has a predominant effect on the dissolution process as it is found in close contact with the hydroxyl groups, while the cation tends to form an outer shell.^48^ Several parameters have been used to explain the solubility of molecules in ILs. It is commonly reported that cellulose solubilization takes place when the hydrogen-bond basicity (HBB), corresponding to β in the Kamlet–Taft system, surpasses 0.95.^20,48^

Since the HBB is primarily influenced by the anion, we have used theoretical data of the anion’s HBB (AHBB) published by Cho et al.^33^ and have selected five ILs displaying large AHBB for experimental screening. The selected ILs and their AHBB are: [Bmim][Ac], 4.84; [Emim][DBP], 4.36; [Bmim][DBP], 4.36; [Bmim][Cl], 4.25; and [Ch][Cl], 4.25. As a comparison, the theoretical AHBB for the anions [Cl] and [N(CN)_2_] are 4.25 and 1.83, while their corresponding β values are 0.65 and 0.95, respectively.^20^

**Figure 3:**
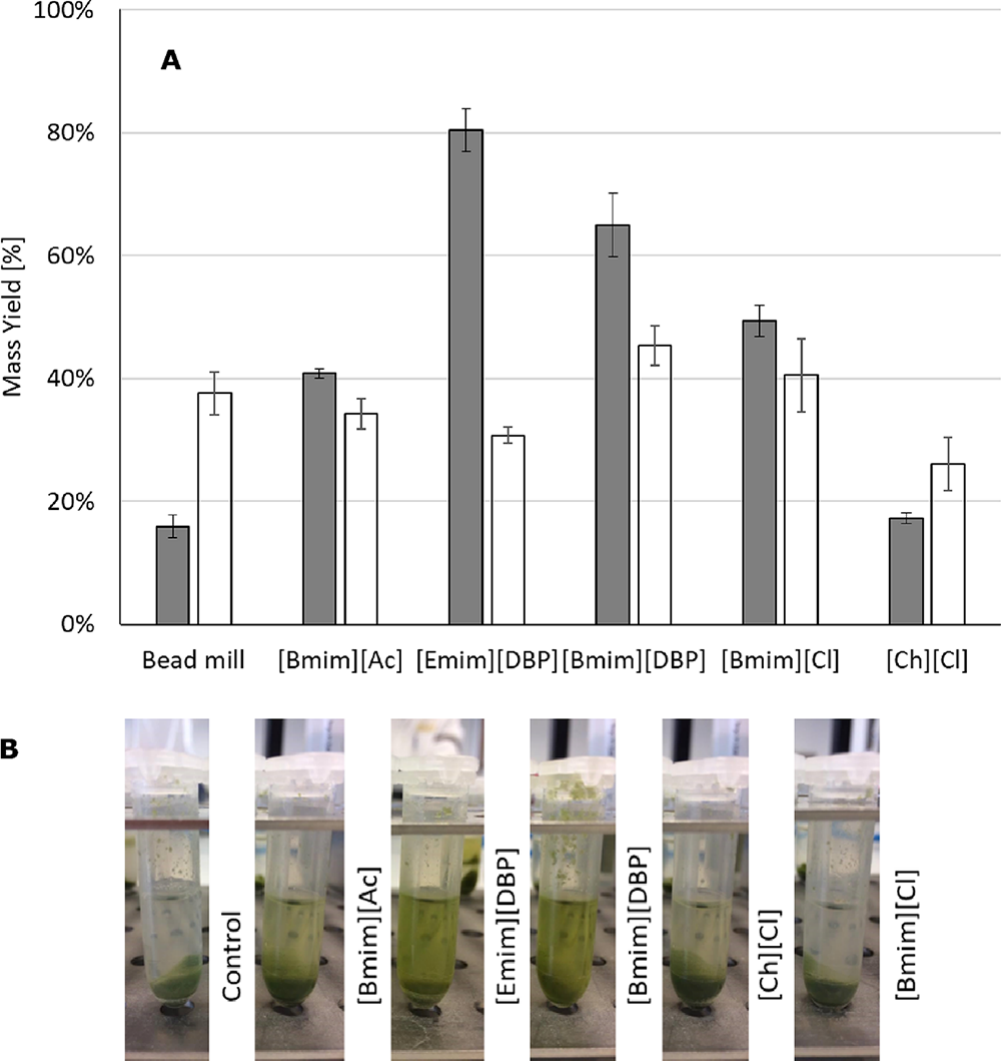
(A) Extraction yields for proteins (gray bars) and carbohydrates (white bars) in samples after treatment with ILs (40 wt %) and bead milling. Data correspond to average values, and corresponding standard deviations are shown as error bars (*n* = 3, experiments conducted in triplicate). (B) Experimental samples of algal biomass treated with ILs. Extraction conducted at room temperature and *t* ≈ 10 min.

The experimental extraction yields are presented in Figure 3A. The extraction experiments with ILs were conducted under bead milling, for which a synergistic effect of mechanical shear and chemical interactions is expected. Figure 2A showed that aqueous extraction yielded only ∼10% of the proteins and ∼20% of the carbohydrates initially present in the biomass, while shear extraction did not enhance the extraction levels. Under bead milling, however, the extraction yields increased almost two-fold (Figure 3A). This can be due to a more efficient contact and shearing effect of the colliding beads with the algal biomass in comparison to the rotor-stator system. The intense mixing generated during bead milling can also contribute to a deeper access of the ILs into the macroalgal structure, favoring the dissolution process.

Significant differences were observed for the extraction of proteins by ILs (Figure 3A). Except for [Ch][Cl], more than 40% of the proteins could be extracted by means of IL-aided shear. A remarkable 80.4% yield was obtained with [Emim][DBP]; in addition, the protein-to-carbohydrate yield ratio is the highest from the series investigated, clearly suggesting a selective extraction ability. The ILs resulting in the highest protein extraction contain the anion dibutyl phosphate [DBP]. This anion has a theoretical hydrophobicity of 0.22, compared to −0.49 for [Cl] and −0.61 for [Ac]. Experimental data also showed that the algal biomass treated with ILs containing [DBP] appeared greener, indicating a higher degree of pigment extraction (Figure 3B). Since most pigments exhibit a nonpolar nature, it is possible that the hydrophobicity of the anion is a key parameter to ensuring high degrees of protein extraction. The intense shear created by the colliding beads and the effect of the ILs in destabilizing hydrogen bonds in the cellulose structure are also contributing to the extraction of proteins rather than the dissolution of cellulose.

The results from Figure 3A clearly point out that the cation of the IL also plays a major role on the extraction of proteins. It has been reported that the smaller length of the alkyl chain and the stronger polar character of the cation results in superior cellulose dissolution.^26^ This appears also to be the case for proteins as the IL containing cations with shorter alkyl chains ([Emim]) led to higher protein solubilizations. Likewise, the theoretical hydrophobicity for the cations of [Emim] and [Bmim] are 0.2 and 0.7, respectively, while that of [Ch] is −0.4. The more hydrophilic character of the [Ch] could be limiting its capacity to solvate proteins from *U. lactuca*.

Only minor differences on the extraction yields of carbohydrates were observed among the ILs tested (Figure 3A). This indicates that no substantial dissolution of cellulose, or other polysaccharide, was achieved by chemical means. The theoretical values of AHBB failed to forecast a significant degree of chemical dissolution beyond what is achieved by shear under aqueous conditions. This can be due to the mild conditions used in the present research: room temperature and 10 min contact time. Most of the published research on the processing of agricultural feedstocks and woody biomass with ILs employ temperatures of 50–190 °C and contact times of 1–72 h.^20^ Hou et al.^49^ reported that 11.5% of microcrystalline cellulose can be solubilized in [Bmim][Ac] at 50 °C, whereas [Bmim][Cl], one the most studied ILs for the processing of biomass, can solubilize 20% at 100 °C.

Temperature is a crucial parameter since it favors the swelling of fibers and destabilizes the hydrogen bonds in the cellulose structure.^50^ No clear dependency could be drawn to relate carbohydrate dissolution to the ILs’ structure or properties.

Pezoa-Conte et al.^24^ investigated the disintegration of *U. rigida* after treatment with three ILs of different chemical natures. The extraction was conducted by directly contacting algal biomass with IL for 6 h and at temperatures in the range of 100–160 °C. A maximum of 67% of the total carbohydrates and up to 42% of the total protein was solubilized by the IL 1,1,3,3-tetramethylguanidine propionate. It is not surprising that the extraction yields for carbohydrates are notably superior compared to our findings, considering the severe conditions in which the extraction was conducted and the strong basic character of the tetramethyl-guanidine cation. Under such conditions, it is also expected that denaturation and hydrolysis of the extracted proteins and labile biomolecules will occur. Malihan et al.^25,26^ studied the dissolution of sugars from the macroalgae *Gelidium amansii* by several ILs. Samples were treated at 120 °C, resulting in sugar yields of 50% using [Bmim][Cl] and 67% using [Tri-EG-(MIm)_2_]_2_[HSO_4_]. None of the published studies addresses the extraction of proteins and the influence of the processing conditions on their native conformation.

In this work, IL [Emim][DBP] was selected for further experiments as it resulted in the highest extraction yields for proteins.

### Effect of the Ionic Liquid Dose

**Figure 4:**
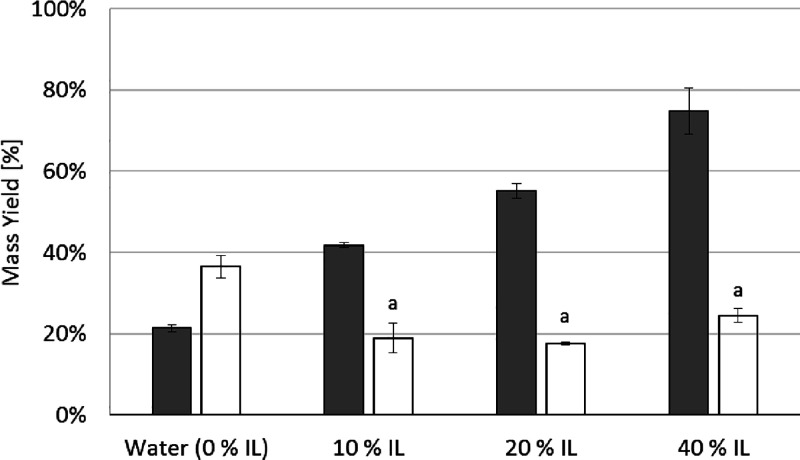
Effect of the concentration of [Emim][DBP] on the extraction of proteins (gray bars) and carbohydrates (white bars). The data corresponds to averages, and the errors bars are the standard deviation (*n* = 2). Lowercase letters indicate statistically equal means at 95% confidence.

Besides temperature and contact time, the IL dose is an important parameter that affects both the extraction yields and the process economics. In the studies conducted by Malihan et al.,^25,26^ the ratio of algal biomass to IL was 3–20 to 100, while Pezoa-Conte et al.^24^ employed a ratio of 11 to 100 and direct contact of the IL with the biomass. In Figure 4 the extraction yields for proteins and carbohydrates are presented for solutions containing 0–40 wt % [Emim][DBP], which corresponds to biomass to IL ratios of 0, 2.5, 5, and 10 to 100. Higher IL concentrations could not be reliably studied due to their high viscosity and problems with the analytical procedures. Within the studied range, there is a direct proportion between the concentration of IL and the protein yields. Similar tendencies were noted by Malihan et al.,^25^ who investigated biomass-to-IL ratios of 3–20 to 100. The authors observed that sugar yields from the macroalga *G. amansii* increase proportionally to the IL content. Our results, on the contrary, show that the extraction of carbohydrates does not vary significantly (*p* > 0.05). According to the experimental data, there is a superior solvation of the biomass at higher concentrations of IL but, due to the superior affinity of the [DBP] for the proteins, only the proteins are solubilized. From Figure 4, it is also evident that [Emim][DBP] leads to a decrease in the extracted amount of carbohydrates. This can be due to the hydrophobicity of the anion, which forces the carbohydrates to remain in the residual insoluble biomass.

### Phase Partitioning

**Figure 5:**
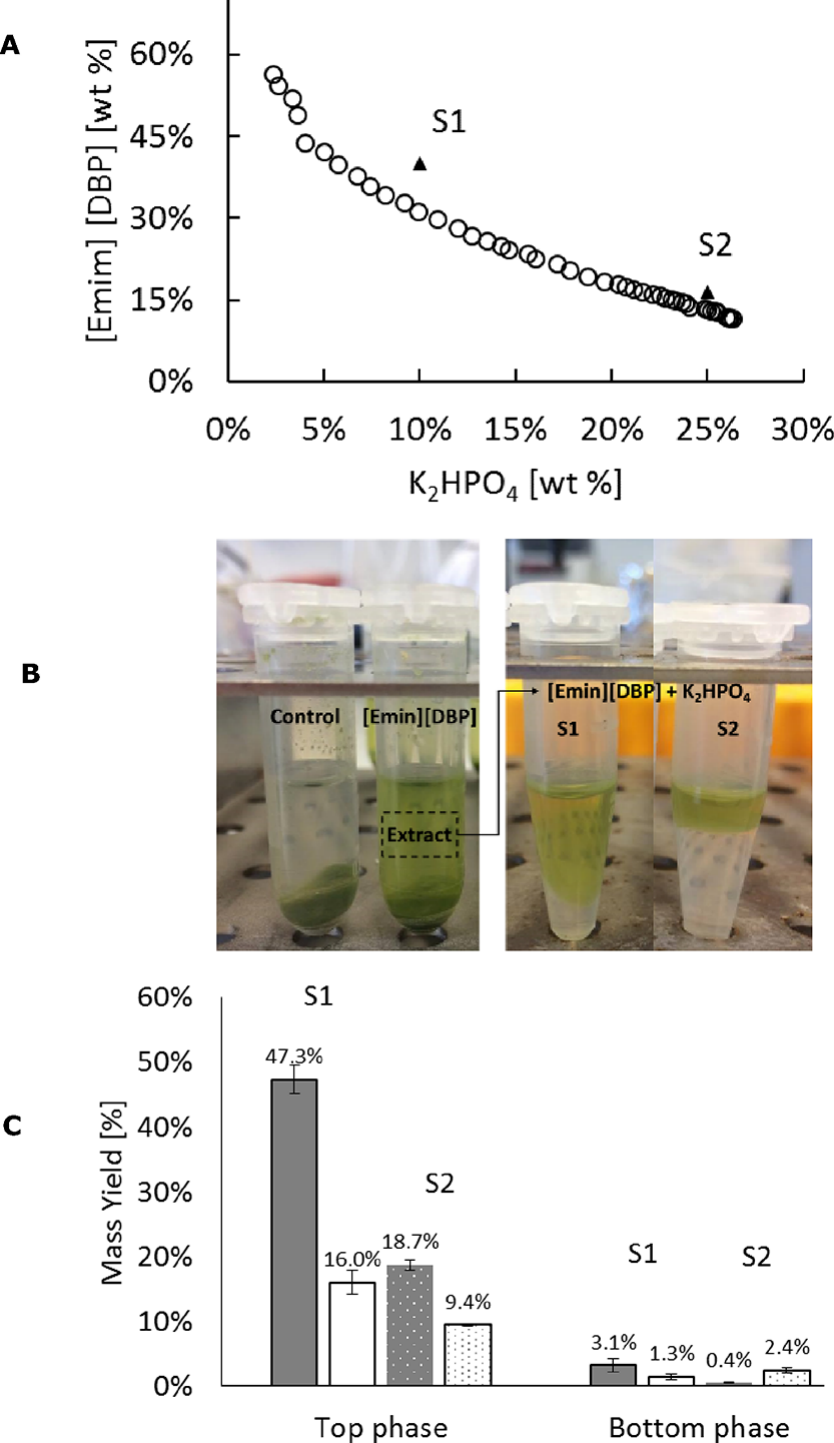
(A) Phase diagram for the system [Emim][DBP]-K_2_HPO_4_ with detail of the operation points S1 and S2. (B) Algal samples treated with [Emim][DBP] followed by the addition of K_2_HPO_4_ for phase induction and biomolecule partitioning. (C) Overall yields for protein (gray bars) and carbohydrates (white bars) after phase formation and partitioning. Data presented here are the average values and errors bars as standard deviation (*n* = 2). Dotted bars are assigned for system S2.

The partitioning of molecules extracted by [Emim][DBP] from *U. lactuca* was studied in two-phase systems formed with K_2_HPO_4_. This salt has been broadly studied in several IL systems for the induction of phase formation aimed at biomolecule fractionation since It allows a greater immiscibility region in the phase diagram.^51^ According to the Hofmeister series,^52^ K_2_HPO_4_ has a moderate to strong protein salting-out character, which is expected to favor its affinity for carbohydrates. The experimental phase diagram for the system [Emim][DBP]- K_2_HPO_4_ is shown in Figure 5A. Two operation points were selected for the partitioning study namely S1 (40% IL, 10% Salt) and S2 (15% IL, 25% salt), which fall within the ranges of IL studied in the previous section.

In Figure 5B, the corresponding algal extract and the two-phase systems are shown. As expected, visual inspection on the samples confirmed that virtually all the pigments remain in the IL-rich top phase. Moreover, quantification experiments showed that nearly 63.0 ± 2.9% of the proteins and 79.9 ± 9.4% of the carbohydrates initially present in the extract remained in the top-phase of system S1 after two-phase formation. For system S2, 41.5 ± 1.8% of the proteins and 47.0 ± 0.9% of the carbohydrates were found in the top phase. Further analysis revealed that 4.2 ± 1.3% of the proteins and 6.7 ± 2.1% of the carbohydrates migrated to the bottom phase of system S1, while for system S2, only 1.0 ± 0.2% of the proteins and 12.1 ± 2.3% of the carbohydrates were transferred to the bottom phase. The corresponding overall yields, taking into account extraction and phase partitioning, are shown in Figure 5C. As can be seen, system S1 leads to the largest protein and carbohydrate recoveries but requires higher concentrations of IL. Although the extraction yields in the bottom phase are substantially low, such molecules are pigment-free, which may be interesting for specific market applications.

Contrary to our expectations and to published studies,^21^ the induction of a bottom salt-rich phase did not cause a significant partitioning of proteins and carbohydrates. This can be due to the nature of the proteins extracted, which may be present within macromolecular assemblies or cross-linked via disulfide bonds to polysaccharides.^32^ Example of such proteins found in *Ulva* sp. are lectins, a structurally diverse group of carbohydrate-binding proteins.^12^ In this respect, proteins and carbohydrates extracted by [Emim][DBP] cannot be separated by means of hydrophilicity differences created by the two-phase system [Emim][DBP]-K_2_HPO_4_ as they may be forming macromolecular complexes.

The experimental yields for systems S1 and S2 reveal that 30–55% of the proteins and 14–40% of the carbohydrates are transferred to a third “insoluble” layer. Visual inspection on the experimental samples confirmed the presence of a subtle interphase, which was clearly noted in experiments with higher biomass concentrations (data not shown). Three-phase partitioning (TPP) was first reported 1984 and has been implemented for the fractionation and purification of proteins^53^ and carbohydrates.^54^ The third layer is usually a precipitate formed between the top and bottom phases. The extent of precipitation and the location of the third layer depends on the salting-out character of the phase-forming components, their density, and the bounds that might be forming with the precipitated molecules.^53^ Although TPP is interesting for purification and concentration purposes, the activity and stability of the molecules precipitated at the interphase may be compromised.

### Recyclability of [Emim][DBP] and Fractionation by Ultrafiltration

The potential application of [Emim][DBP] in the biorefinery of *U. lactuca* at commercial scale depends not only on its ability to selectively extract biomolecules under mild conditions and high yields but also on its recovery. In addition, the extracted biomolecules must be separated from the IL in order to allow their use as a marketable product. In Table 1, the mass yields for proteins, carbohydrates, and [Emim][DBP] are presented for two different membrane configurations: 10 kDa poly-ether sulfone (PES) and 3 kDa regenerated cellulose (R.Cell). In the control samples for both configurations, no carbohydrates were detected in the retentate or permeate phases (data not shown), indicating that the IL did not lead to a significant solubilization of the membranes’ polysaccharides.

**Table 1:** Mass Yields for Biomolecules and IL after Filtration of Algal Extracts^b^

		proteins (%)	carbohydrates (%)	[Emim][DBP]^a^ (%)
PES	retentate	71.3 ± 8.2^a^	85.4 ± 8.6	14.6 ± 0.7
	permeate	6.2 ± 2.1	6.2 ± 2.2	85.2 ± 6.0^A^
R.Cell	retentate	80.4 ± 3.3^a^	50.0 ± 7.9	20.4 ± 0.5
	permeate	0.3 ± 1.2	2.4 ± 0.2	79.6 ± 3.3^A^

a Samples measured in duplicate.

b Data are the average values and corresponding standard deviations. Capital and lowercase letters indicate statistically equal means at 95 % confidence interval for permeates and retentates, respectively.

The experimental data show that the proteins and carbohydrates are mostly recovered in the retentate phase, while the IL migrates preferentially to the permeate phase. Visual observations indicate that pigments are retained in the retentate phase (data not shown). The superior recovery of biomolecules in the permeate is due to the low cut-offs and hydrophilic nature of the materials in the PES and R.Cell membranes. The fact that most of the IL is present in the permeate suggest that the IL is not forming macromolecular complexes with either proteins or carbohydrates.

Although there are no statistical differences in the performance of the membranes for retaining proteins or filtering IL, the retention of carbohydrates showed dependency. Higher carbohydrate yields were measured in the PES membrane, which has a larger cut-off (10 kDa), suggesting that the material of the R.Cell membrane (regenerated cellulose) presents a lower degree of interaction with the algal polysaccharides and thus allowing their transport across the membrane.

From the data in Table 1, it can be deduced that ∼20% of the proteins and ∼10–40% of the carbohydrates are lost, probably as result of their precipitation on the membrane’s layer. Membrane fouling is a common phenomenon that negatively impacts the filtration performance and leads to biomolecule losses, as reported for microalgae.^55^ To the authors’ knowledge, this is the first study in which ultrafiltration is investigated for the fractionation of biomolecules from macroalgae and to recover ILs from algal extracts. The only reports about filtration technology in macroalgae are related to the use of diafiltration to remove the excess of salts after protein precipitation with (NH_4_)_2_SO_4_.^56,57^

### Stability of Proteins in Ionic Liquid Extracts

**Figure 6:**
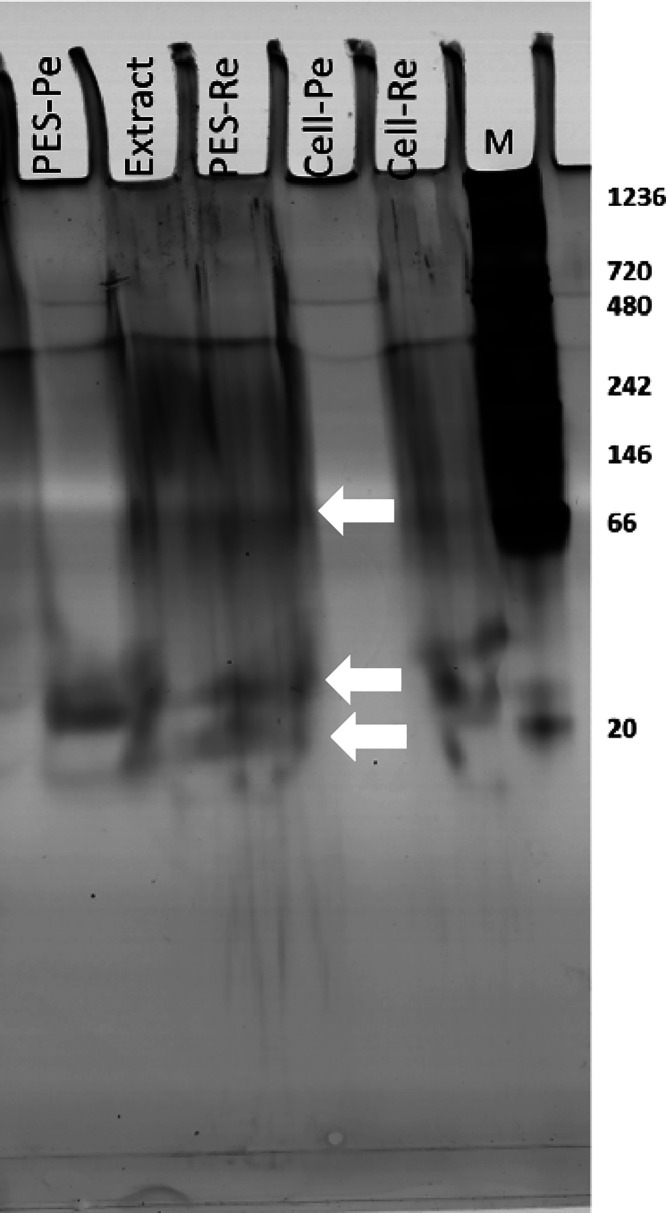
Native gel electrophoresis of samples before and after filtration studies (Pe: Permeate, Re: Retentate, M: Marker; white arrows indicate main protein bands).

ILs are reported to provide a mild environment that favors protein stability and activity. However, at high concentrations, it can lead to molecular aggregation and loss of native conformation.^21,58^ In this regard, native-gel analysis was conducted to evaluate the effect of the extraction and filtration steps on the algal proteins. The results shown on Figure 6 indicate that the proteins extracted by [Emim][DBP] remained mostly in their native conformation as the protein bands are comparable to the protein bands observed after aqueous extraction (Figure 2B). As expected, the permeate of the PES membrane is rich in low-molecular weight proteins (∼20 kDa), while large-molecular weight proteins are present in the retentates. In accordance with the yields presented in Table 1, no bands were observed for the permeate of the R.Cell membrane, suggesting that this fraction only contains small peptides and amino acids.

### Outlook

**Figure 7:**
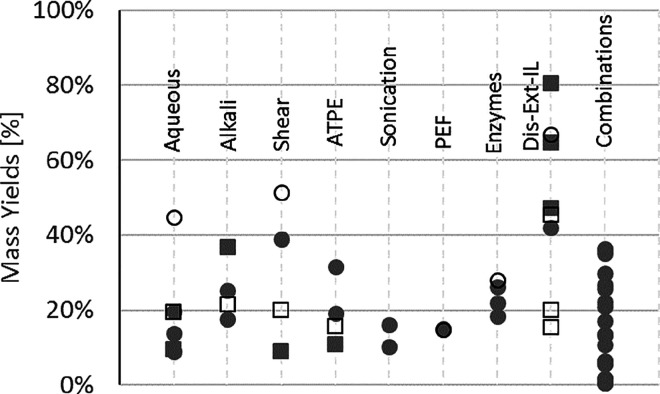
Overview of processes for the recovery of proteins (filled markers) and carbohydrates (open markers) from green macroalgae. Circles represent published data, and squares represent data from the present study.

We have presented an IL-mediated process for the extraction of biomolecules from the macroalga *U. lactuca* and two approaches to achieve partitioning: induced phase formation and ultrafiltration. With this approach, we reached overall extraction yields of 80.4% for proteins with [Emim][DBP] and 45.4% for carbohydrates using [Bmim][DBP]. Furthermore, after phase induction and filtration, the overall yields decrease as a result of losses at the interphase and membrane layer. The experimental yields obtained in this investigation were compared with several studies in which the biorefinery of green macroalgae was investigated. The results are plotted in Figure 7.

All the reported processes resulted in protein extractions below 42%, while the maximum reported carbohydrate yields is 67%.^24^ Besides the conventional processes discussed in Extraction of biomolecules with conventional methods, several authors have investigated physical methods such as ultrasound,^15^ pulsed electric fields,^17,59^ and combinations of technologies. Kandasamy et al.^56^ and Wong and Cheung^57^ evaluated a complex set of unit operations including osmotic shock, aqueous extraction (35 °C), alkali hydrolysis (pH 12), precipitation ((NH_4_)_2_SO_4_), and dialysis to extract proteins from three strains of the genus *Enteromorpha* and *U. lactuca*. The extraction yields varied considerably from 5.7 to 36%. Amano and Noda (1992)^18^ used sequences of extractions including enzyme hydrolysis, buffer extraction, alkali extraction (pH 12), and precipitation with trichloroacetic acid on protoplasts and thallus of strains of the genus *Ulva* and *Enteromorpha.* The reported yields ranged from 0.5 to 35%. Sonication^15^ and pulsed electric fields^17,59^ in general led to poor yields, which is expected given the complexity and toughness of the studied macroalgal biomass. Such diverse range of yields reflect not only the influence of the process but also on the physiology and composition of the algal strain.

With the IL-assisted process presented here “disruption–extraction–ionic liquid” (Dis-Ext-IL), a maximum of 80% of proteins can be recovered in a single step, 47% can be recovered in the top phase after phase induction, and nearly 65% can be recovered in the retentate after filtration. Despite the promising results obtained for [Emim][DBP], several aspects still require comprehensive investigation. The mechanism of extraction is far from being elucidated, and the structural effects on the algal biomass require further understanding. A more complete analysis of the extraction processes is needed in order to quantify the purity of the biomolecules, including characterization of the interphases and determination of the extraction yields for ash and pigments. Moreover, studies on the reusability of the IL are pending in order to learn if the IL retains the extraction capabilities after its recovery via filtration. The crude alga extract (retentate after filtration) is an IL-poor stream containing most of the algal biomolecules. However, it still has significant amounts of IL, making it unsuitable for commercial products. Additional research is needed in order to develop processes that allow the production of IL-free extracts and to characterize their composition, stability of biomolecules, and techno-functional properties.

## Conclusions

In this investigation, we report the selective extraction of biomolecules from the green macroalgae *U. lactuca*. It was demonstrated that the extraction process, mediated by ionic liquids, is selective to proteins and can be conducted under mild conditions. The extraction yield of proteins reached a maximum of 80.4% with ethyl methyl imidazolium dibutyl phosphate [EMIM][DBP], which was attributed to the hydrophobicity of its anion. Biomolecule partitioning was proven by means of two-phase induction. However, the fractionation performance was poor as proteins and carbohydrates were not effectively fractionated. This suggested that proteins and carbohydrates form macromolecular complexes, and thus, they cannot be separated by means of phase partitioning. Similarly, the separation of biomolecules and ionic liquid was investigated utilizing ultrafiltration. It was found that proteins and carbohydrates are preferentially recovered in the retentate phase, while up to 85.2% of the ionic liquid migrates to the permeate phase. In overall, up to 64.6% of the proteins and 15.4% of the carbohydrates were found in the retentate phase after extraction and filtration. The protein yields are notably superior compared to other extraction–fractionation processes reported in the literature.
